# SPARK: A webserver for comprehensive search and analysis of testicular single‐cell transcriptomic data covering full lifecycle and pathology

**DOI:** 10.1002/imo2.70073

**Published:** 2026-01-10

**Authors:** Lina Cui, Xiaojian Lu, Hui Cai, Ziyan Zhuang, Yifei Guo, Qiaoling Jiang, Zhe Zhang, Hui Jiang, Xiaoyan Wang, Jingtao Guo

**Affiliations:** ^1^ State Key Laboratory of Organ Regeneration and Reconstruction, Institute of Zoology Chinese Academy of Sciences Beijing China; ^2^ Beijing Institute for Stem Cell and Regenerative Medicine Beijing China; ^3^ University of the Chinese Academy of Sciences Beijing China; ^4^ Department of Urology, Center for Reproductive Medicine, State Key Laboratory of Female Fertility Promotion Peking University Third Hospital Beijing China; ^5^ Department of Urology Peking University Third Hospital Beijing China; ^6^ Department of Urology, Institute of Urology Peking University First Hospital Beijing China

## Abstract

SPARK is an interactive platform hosting 189,232 human testicular single‐cell transcriptomes across normal and infertile conditions. It enables automated, code‐free analysis and visualization, providing a powerful resource to explore cellular and molecular dynamics in male reproduction and infertility.

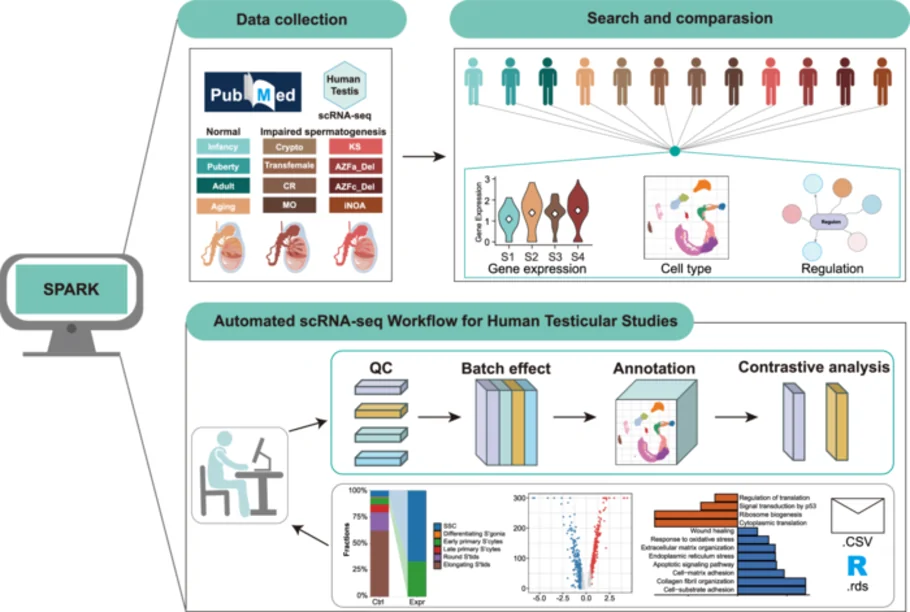

## ETHICS STATEMENT

The study was conducted under ethical approval from the ethics committee of Peking University Third Hospital (IRB00006761‐M2022692).

To the Editor,

The structure and function of the human testis depend on coordinated interactions among diverse cell types. Proper germ cell development is vital for male fertility, but this process can be disrupted by genetic factors—such as Klinefelter syndrome (KS) [1], Y chromosome azoospermia factor (AZF) region microdeletion (AZF_Del) [2], and idiopathic non‐obstructive azoospermia (iNOA) [2, 3, 4], as well as lifestyle factors like smoking [5] and body mass index [6].

Single‐cell RNA sequencing (scRNA‐seq) has transformed our ability to study the human testis by revealing key molecular signatures of normal and impaired spermatogenesis [7, 8]. While recent resources like SMARTdb [9], the Male Healthy Atlas (MHA) [10], and HISTA [11] support testis‐focused analyses, they remain limited in dataset coverage and analytical flexibility, particularly in cross‐dataset comparisons and automated workflows.

To address this, we developed SPARK (Single‐cell Physiological and pathological Analysis of male Reproductive Kinetics), a user‐friendly and comprehensive scRNA‐seq database for human testis. SPARK hosts 189,232 single‐cell transcriptomes from 29 patients and 19 healthy donors, spanning 9 infertility types and normal development. It enables interactive exploration of cell clustering, gene expression, transcriptional regulation, and pathway enrichment—without requiring coding expertise. By integrating robust visualization and analysis tools, SPARK supports deeper insights into male reproductive biology and disease, facilitating broader access to high‐resolution testicular data.

## 1 OVERVIEW OF SPARK

SPARK is a comprehensive and interactive web server designed to advance research on human male infertility and normal spermatogenesis through single‐cell transcriptomic data. The platform is organized into three main sections. The first section allows for individual processing and analysis of single‐cell transcriptomic data across various groups, including 51 donors, 17 datasets, 189,232 cells, 9 infertility conditions, and 24 distinct testicular cell types (Figure 1A, Supporting Information S1: Figure S1, and Supporting Information S1: Table S1). Users can explore cell type distributions within each group, assess gene expression levels, and identify specific markers associated with each cell type (Supporting Information S1: Movie S1). The second section features an atlas that encompasses normal developmental stages (infant, puberty, adult, and aging) and conditions of impaired spermatogenesis (e.g., cryptorchidism [CR], Mumps orchitis, cryptozoospermia, transgender, KS, AZFa deletion [AZFa_Del], AZFc deletion [AZFc_Del], and iNOA influenced by genetic and environmental factors (Figure 1B and Supporting Information S1: Figure S2). This atlas enables researchers to investigate molecular dynamics throughout spermatogenesis and compare differentially expressed genes (DEGs) between healthy and diseased individuals (Figure 1C and Supporting Information S1: Movie S2). Additionally, the platform provides insights into transcription factors (TFs) identified using pySCENIC, showcasing variations in gene regulation and expression across different groups within the male testes (Figure 1D).

**Figure 1:**
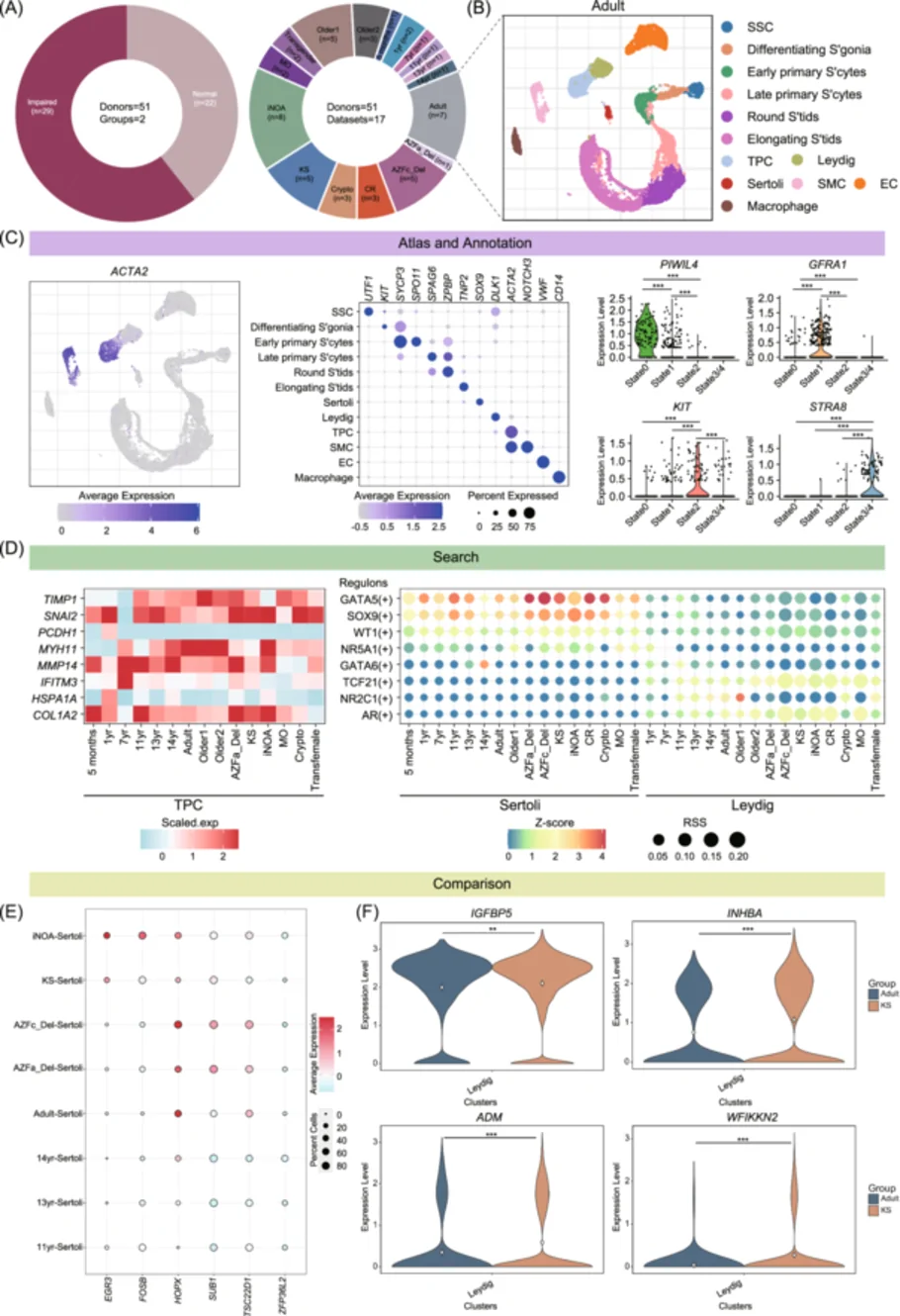
Function of SPARK. (A) The diagram showing the distribution of spermatogenesis types (left) and data source (right). AZFa/c_Del, Y chromosome azoospermia factor A/C region microdeletion; CR, cryptorchid; Crypto, cryptozoospermia; iNOA, idiopathic nonobstructive azoospermia; KS, Klinefelter syndrome; MO, mumps orchitis; yr, years old. (B) UMAP plots showing the distribution of different datasets in human testes. EC, endothelial cell; S'gonia, spermatogonia; S'cytes, spermatocytes; SMC, smooth muscle cell; SSC, spermatogonial stem cells; S'tids, spermatids; TPC, testicular peritubular cells. (C) Left: UMAP plot showing the expression of *ACTA2* in adult males. Middle: Dot plot showing the expression of markers in the adult males. Right: violin plots showing the normalized expression of *PIWIL4*, *GFRA1*, *KIT*, and *STRA8* in the spermatogonia. Each black point represents the normalized expression in an individual cell. Pairwise group comparisons were performed using the two‐sided Wilcoxon rank‐sum test. For all pairwise comparisons, post hoc tests were conducted using the two‐sided Wilcoxon rank‐sum test without Bonferroni correction. ***p < 0.001. (D) Left: heatmap plot showing the scaled expression of genes in TPCs in different groups. Right: bubble plot showing the enriched regulons of Sertoli and Leydig cells in different groups. TPC, testicular peritubular cells. (E) Dot plots showing the expression of *EGR3, FOSB, HOPX, SUB1, TSC22D1*, and *ZFP36L2* in different groups. (F) Violin plots showing the expression of *IGFBP5, ADM*, and *INHBA* in KS and healthy samples. KS, Klinefelter syndrome. Two‐sided Wilcoxon rank‐sum test. **p < 0.01, ***p < 0.001.

Furthermore, we have conducted differential gene expression analysis to highlight differences between males with infertility and healthy individuals. These results are accessible on the website, allowing users to visualize genes of interest through MA plots (Supporting Information S1: Figure S3A), violin plots (Supporting Information S1: Figure S3B), and heatmaps. For example, our analysis shows increased expression levels of *EGR3*, *FOSB*, and *SUB1* in Sertoli cells from individuals with Klinefelter syndrome, alongside decreased expression of *HOPX* compared to healthy males, consistent with previous research [2] (Figure 1E). Additionally, we observed overexpression of *ADM*, *INHBA*, *WFIKKN2*, and *IGFBP5* in Leydig cells from KS patients, aligning with findings documented in the literature [12] (Figure 1F). These results are well‐matched with published studies, demonstrating the reliability and validity of our web server. Users can also download these results for further personalized analyses, such as Gene Ontology and KEGG pathway analyses, using online tools like DAVID [13] and Metascape [14].

Recognizing the complexities of scRNA‐seq analysis, we have developed a user‐friendly platform designed to streamline the analysis of human testis scRNA‐seq data and facilitate the export of results (Figure 2A, Supporting Information S1: Figure S4, and Supporting Information S1: Movie S3). This platform simplifies the process by requiring only the gene expression matrix or the three downstream files from Cellranger (10× Genomics). Upon uploading, the platform processes the data and generates visual summaries of the results.

**Figure 2:**
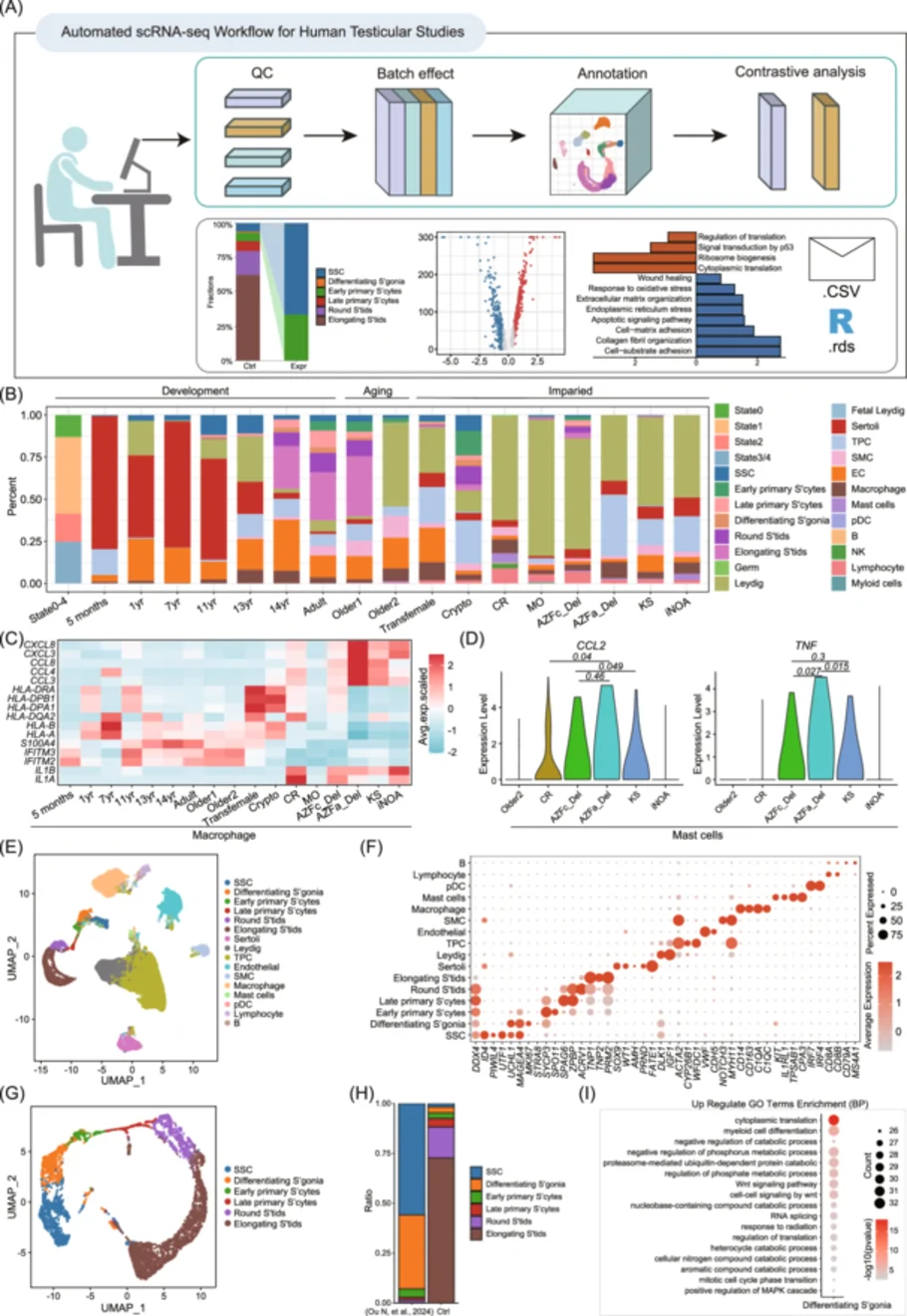
Examples of SPARK. (A) The process of analysis pipeline for human testicular scRNA‐seq datasets in the SPARK. (B) Bar graph showing the distribution of testicular cell types in each group. AZFa/c_Del, Y chromosome azoospermia factor A/C region microdeletion; B, B cell; CR, cryptorchid; Crypto, cryptozoospermia; EC, endothelial cell; iNOA, idiopathic nonobstructive azoospermia; KS, Klinefelter syndrome; MO, mumps orchitis; NK, natural killer cell; pDC, plasmacytoid dendritic cell; S'cytes, spermatocytes; SSC, spermatogonial stem cells; S'gonia, spermatogonia; S'tids, spermatids; TPC, testicular peritubular cells; SMC, smooth muscle cell; yr, years old. (C) Heatmap plot showing the expression of representative genes in each group in macrophages. (D)Violin plots showing the expression of *CCL2* and *TNF* in mast cells in different groups. Pairwise group comparisons were performed using the two‐sided Wilcoxon rank‐sum test. For pairwise group comparisons, post hoc tests were conducted using the two‐sided Wilcoxon rank‐sum test without Bonferroni correction. (E) UMAP showing the distribution of clusters in AZFc_Del patients. (F) Bubble plot showing the expression of marker genes. (G) UMAP showing the distribution of germ cells in AZFc_Del patients. (H) Bar plot showing the distribution between AZFc_Del patients (Ou et al., 2024) and healthy donors (Ctrl). (I) Dot plot showing the GO enrichments in differentiating spermatogonia, when comparing AZFc_Del to healthy control samples.

SPARK serves as an interactive and intuitive tool for visualization and analysis, addressing a wide range of human infertility diseases and subfertility types. Its goal is to deepen understanding of nonobstructive azoospermia and identify potential targets, including specific cell types and genes relevant to various forms of subfertility.

## 2 CASE STUDY ON SPARK

Upon reviewing the database, we noted an increased presence of immune cells—such as macrophages, mast cells, lymphocytes, NK cells, and B cells—in impaired and abnormal testicular samples (Figure 2B). Macrophages, which play essential roles in both immune and nonimmune functions within mammalian male gonads [15, 16], displayed distinctive gene expression patterns related to antigen presentation and cytokine production. Existing research indicates that macrophages secrete cytokines involved in inflammatory responses and interact with various testicular cells, including Leydig cells [17], endothelial cells, Sertoli cells, and germ cells. Notably, genes such as *CCL3*, *CCL4*, *CCL8*, *CXCL3*, and *CXCL8* were found to be upregulated in conditions like CR, AZFa_Del, AZFc_Del, KS, and iNOA (Figure 2C).

We also observed a negative correlation between the number of mast cells and KI67+ spermatocytes [16]. *CCL2* emerged as a potential biomarker linked to mast cell infiltration in the testicular immune microenvironment during spermatogenic dysfunction [18]. Consequently, visualizations of *CCL2* and *TNF‐α* in mast cells underscore their involvement in immune activation and spermatogenic dysfunction (Figure 2D).

Additionally, we used a published dataset on AZFc_Del from Ou et al. [19] to showcase the capabilities of our analysis module. After uploading the gene expression matrix and selecting a control sample for comparison, we were able to visualize and download the results within about an hour. Our initial outputs included uniform manifold approximation and projection (UMAP) visualizations (Figure 2E), marker expression profiles (Figure 2F), and a summary of cell ratios, revealing that AZFc_Del samples had a higher proportion of spermatocytes and a lower proportion of spermatids (Figure 2G,H), consistent with the findings described in the literature [19]. Moreover, the original study identified DEGs from AZFc_Del samples compared to obstructive azoospermia controls in c‐KIT‐positive spermatogonia, which were enriched in “cytoplasmic translation” and “mitotic cell cycle phase transition” pathways [19]. Using our analysis pipeline, we similarly detected these pathways in differentiating spermatogonia when comparing AZFc_Del samples to those from healthy control (Figure 2I). These results closely match those reported in the original study [19], demonstrating the robustness of our analysis pipeline.

SPARK is a centralized and interactive platform designed for comprehensive analysis of human testicular single‐cell transcriptomes. It integrates 189,232 cells from 51 samples covering nine infertility conditions as well as normal developmental stages, providing an unprecedented resource for exploring the cellular and molecular landscape of the testis. Notably, SPARK combines rare clinical datasets, rigorous data harmonization, and intuitive analysis modules within a single accessible platform.

The platform incorporates both published and newly sequenced datasets, including conditions not previously represented such as mumps orchitis. Each dataset undergoes rigorous quality control and standardized preprocessing steps such as normalization, batch effect correction, and dimensionality reduction, ensuring consistency across studies. This integration enables direct comparisons between healthy and diseased testes across developmental stages and allows the identification of both shared and condition‐specific mechanisms underlying spermatogenic failure.

Compared to existing platforms (Supporting Information S1: Table S2), SPARK offers broader data coverage, richer disease representation, and streamlined analytical workflows. Its interactive modules allow users to query gene expression, explore regulatory networks, and perform cross‐condition analyses. The integrated analysis pipeline further lowers the technical barrier by allowing researchers without bioinformatics expertise to process their own data from input to visualization within a validated framework. This dual role as repository and discovery tool ensures that SPARK not only replicates published findings but also facilitates new insights into infertility mechanisms, including immune cell dynamics and regulatory changes in testicular function.

Looking forward, SPARK will expand to integrate multi‐omics data, cover additional infertility types, and accept community‐contributed datasets. Together, these advances will establish SPARK as a continuously evolving ecosystem that lowers technical barriers, accelerates research in testicular biology, and informs the development of diagnostic and therapeutic strategies for male infertility.

## AUTHOR CONTRIBUTIONS

**Lina Cui**: Writing—original draft; investigation; methodology; formal analysis; data curation; conceptualization. **Xiaojian Lu**: Investigation; writing—original draft; methodology; formal analysis; data curation; conceptualization. **Hui Cai**: Data curation; **Ziyan Zhuang**: Data curation. **Yifei Guo**: Methodology. **Qiaoling Jiang**: Methodology. **Zhe Zhang**: Resources; Supervision. **Hui Jiang**: Resources; supervision. **Xiaoyan Wang**: Writing—review and editing; project administration; conceptualization; methodology; supervision. **Jingtao Guo**: Writing—review and editing; project administration; conceptualization; methodology; supervision. All authors have read the final manuscript and approved it for publication.

## CONFLICT OF INTEREST STATEMENT

The authors declare no conflict of interest.

## Supporting information

Supporting information.

Supporting information.

Supporting information.

**Supporting Information to SPARK:** A webserver for comprehensive search and analysis of testicular single‐cell transcriptomic data covering full lifecycle and pathology.

**Table S1.** Detailed data information included in this study.
**Table S2.** Comparative information among SPARK and other existing platforms such as SMARTdb, MHA, and HISTA.

## Data Availability

The published data have been downloaded from GEO (GSE182786 for aging datasets; GSE154535, GSE149512 and GSE169062 for NOA; GSE130151, GSE149512 and GSE169062 for KS; AZFa_Del download from GSE149512; GSE153947 and GSE134144 for Cryptozoospermia and Transgender, respectively; GSE134144, GSE161617 and GSE120508 for fetal testis) and the raw data of AZFc_Del, Mumps orchitis and CR have been deposited in the GSA‐human (Mumps orchitis: https://ngdc.cncb.ac.cn/gsa-human/browse/HRA006586; AZFc_Del: https://ngdc.cncb.ac.cn/gsa-human/browse/HRA007747; CR: https://ngdc.cncb.ac.cn/gsa-human/browse/HRA006558). SPARK is publicly available at http://spark.guolab.info. Statements of SPARK and code availability are available at https://github.com/JingtaoLab/SPARK. Supplementary materials (methods, figures, tables, graphical abstract, slides, videos, Chinese translated version and updated materials) may be found in the online DOI or iMetaOmics http://www.imeta.science/imetaomics/.
