# Supplementary material for: SPARK: A webserver for comprehensive search and analysis of testicular single‐cell transcriptomic data covering full lifecycle and pathology
Source: IMetaOmics. 2026 Jan 10;3(2):e70073. doi: 10.1002/imo2.70073 (PMC13674385; doi:10.1002/imo2.70073)
Supplement: Supplementary file 1 [file IMO2-3-e70073-s001.docx]

**Supporting information to** **SPARK: a webserver for comprehensive search and analysis of testicular single-cell transcriptomic data covering full lifecycle and pathology**

**Running title:** SPARK: Testicular Single-Cell Transcriptomic Webserver for Lifecycle and Pathology

Lina Cui^1,2,#^, Xiaojian Lu^1,2,#^, Hui Cai^1,2,3^, Ziyan Zhuang^1,2,3^, Yifei Guo^1,2,3^, Qiaoling Jiang^1,2,3^, Zhe Zhang^4,5,*^, Hui Jiang^5,6,*^, Xiaoyan Wang^1,2,*^, Jingtao Guo^1,2,3,*^

^1^State Key Laboratory of Organ Regeneration and Reconstruction, Institute of Zoology, Chinese Academy of Sciences, Beijing, 100101, China

^2^Beijing Institute for Stem Cell and Regenerative Medicine, Beijing, 100101, China

^3^University of the Chinese Academy of Sciences, Beijing, 100049, China

^4^State Key Laboratory of Female Fertility Promotion, Center for Reproductive Medicine, Department of Obstetrics and Gynecology, Peking University Third Hospital, Beijing, 100191, China

^5^Department of Urology, Peking University Third Hospital, Beijing, 100191, China

^6^Department of Urology, Institute of Urology, Peking University First Hospital, Beijing, 100034, China

#These authors contributed equally: Lina Cui, Xiaojian Lu

*Correspondence: [jingtao.guo@ioz.ac.cn](mailto:jingtao.guo@ioz.ac.cn) (Jingtao Guo);

[xyan.wang@ioz.ac.cn](mailto:xyan.wang@ioz.ac.cn) (Xiaoyan Wang);

[jianghui@bjmu.edu.cn](mailto:jianghui@bjmu.edu.cn) (Hui Jiang);

[zhezhang@bjmu.edu.cn](mailto:zhezhang@bjmu.edu.cn) (Zhe Zhang)

**Section A: detailed descriptions for SPARK modules**

**Home and Atlas Page**

The Home page provides an overview of the database’s contents through a scrolling diagram that visually represents sample information, spermatogenesis processes, and cell type composition across different testicular states. Users can access introductory information about the database and navigate the functions of each section. The page also includes links to related websites for additional analyses, along with updated news and citation information.

On the Atlas page, users can select datasets to examine the distribution of cell types and gene expression. An interactive uniform manifold approximation and projection (UMAP) plot shows the distribution of testicular cell types, allowing users to focus on specific types of interest. Additionally, UMAP and violin plots illustrate the expression patterns and levels of specific markers and genes within each cell type. For further analysis, a searchable and downloadable table is available, enabling users to validate findings and perform detailed investigations.

**Search Page**

This interface includes three modules designed to offer intuitive access to features related to human testicular development and to compare differences between infertility states according to user-specific needs.

In the "Query Gene Expression" section, users can search for datasets, cell types, and genes using single or multi-selection options. Selecting a gene will generate a violin plot showing its expression levels across chosen cell types and datasets, with each dataset represented by a different color. For multiple genes, expression levels are visualized through dot plots and heatmaps across selected datasets and cell types. Users can also adjust image sizes using the "Plot Size" option to suit various viewing preferences.

The "TF (Transcription factor) Regulons Visualization" module allows users to explore variations in transcription factor regulon expression among different groups. This module functions similarly to the "Query Gene Expression" section, enabling users to delve into gene regulatory networks based on their specific interests.

In the "Datasets Comparison" module, users can conduct differential gene expression analysis between cell types from any two selected datasets. Cell types can be chosen individually or in batches. The results are displayed using MA plots, and the data is stored in an embedded table for easy reference. Users can select one or multiple genes of interest from the differentially expressed genes (DEGs) table and visualize them using the "visualization" button, which provides violin plots and heatmaps to illustrate the expression patterns of the selected genes.

**Analysis Page**

The Analytics module functions as an automated platform for analyzing single-cell RNA Sequencing (scRNA-seq) data related to human testis research. Users need to submit either a single file or three files containing the raw gene expression matrix. A dataset from healthy males is used as a positive control for data integration. The submitted data undergoes a thorough processing pipeline, including normalization, scaling, principal component analysis (PCA), UMAP visualization, cell type annotation, differential gene expression analysis, and pathway enrichment analysis. This entire process typically takes between 0.5 to 1 hour to complete.

Once processing is finished, users can visualize the patterns in their data and download the results for further personalized analysis. Additionally, users who choose to contribute their dataset to our webserver will have their data integrated into the Atlas and search pages, enhancing the overall scope and detail of the database.

**Section B: Materials and methods**

**Data collection**

We gathered single-cell transcriptome data pertaining to the testis from a range of published studies (**Table S1**), obtaining gene expression matrices from the Gene Expression Omnibus (GEO) hosted by the National Center for Biotechnology Information (NCBI) and the Genome Sequence Archive for Human (GSA-human) [1] in the National Genomics Data Center [2], China National Center for Bioinformation. In addition to datasets covering various physiological developmental stages, we included data on aging, high body mass index (BMI), human infertility, and subfertility. Overall, we curated single-cell data from more than 189,232 high-quality cells across 51 samples, spanning infancy, puberty, adulthood, and aging, as well as individuals affected by conditions such as Mumps orchitis (MO), testosterone suppression (Transgender), cryptorchid (CR), and cryptozoospermia (Crypto). Additionally, we included data on other infertility conditions like idiopathic non-obstructive azoospermia (iNOA), Klinefelter syndrome (KS), AZFa deletion (AZFa_Del), and AZFc deletion (AZFc_Del). The MO and AZFc_Del samples were obtained from individuals undergoing surgical testicular sperm extraction for histological assessment or *in vitro* fertilization. Informed consent was obtained from all patients prior to surgery. This study was approved by the ethics committee of Peking University Third Hospital. These datasets collectively cover the entire lifespan of males (**Table S1**).

**Data processing and clustering**

To visualize cell type distributions and identify significant signatures across diverse samples, we conducted cell clustering and annotation analyses. Initially, we integrated datasets from the same sources and utilized Seurat [3] (v4.3.0) for rigorous quality control. Specifically, we included only cells that met stringent criteria: detection of more than 500 genes per cell, a mitochondrial gene ratio below 25%, and unique molecular identifier (UMI) counts per gene per cell between 800 and 20,000. Following these quality control steps, we performed log normalization on the integrated gene expression matrix, identified the top 2000 highly variable genes (HVGs), and applied scaling. PCA was then employed to assess and address batch effects across different datasets. For datasets showing significant batch effects, we applied the RunHarmony function from the harmony package [4] (v0.1.1) to mitigate these effects, thereby improving subsequent analysis and visualization post-PCA. Next, we utilized the Louvain algorithm to cluster cells, resulting in defined clusters. To visualize these clusters in a reduced dimensional space, we employed UMAP, embedding cells with cluster information for intuitive visualization.

To annotate cell types, we employed a well-annotated dataset and transferred labels to the input dataset using SingleR [5] (v2.0.0). Additionally, we referenced published markers and identified the top 50 genes using the FindAllMarkers function with default parameters (min.pct = 0.1, only.pos = TRUE, logfc.threshold = 0.25, assay = "RNA") to validate the accuracy of cell type assignments. Subsequently, the annotated datasets containing identified cell types were prepared for downstream visualization.

For UMAP visualization, we extracted UMAP embedding information, including cell types, from each dataset. We utilized plotly (v5.9.0) in Python 3 to generate interactive visualizations, enabling users to selectively explore the distribution of specific cell types of interest.

**Differential expression analysis**

To examine distinctions among various groups, we conducted pairwise differential expression analysis using the FindMarkers function within Seurat with default settings (assay = "RNA", only.pos = FALSE, min.pct = 0.1, logfc.threshold = 0.25, ident.1 = group1, ident.2 = group2). The results were then filtered based on a significance threshold of *p* < 0.05.

**SCENIC analysis**

To uncover key transcription factor programs (regulons) and explore regulatory networks, we conducted pySCENIC analysis [6] (v0.12.1). We obtained the human cisTarget database from https://resources.aertslab.org/cistarget and followed a three-step approach: first, converting the expression matrix into loom format. Next, we used the 'pyscenic grn', 'pyscenic ctx', and 'pyscenic aucell' functions to infer the gene regulatory network, predict regulon activity, and identify gene regulatory networks associated with major transcription factors specific to each cell type. We calculated Regulon Specificity Scores (RSS) within each cluster and exported these results for visualization.

**Visualization of gene expression levels**

To visualize the distinct expression profiles of each cell type, we converted Seurat objects into anndata objects using SeuratDisk (v0.0.0.9020). The data were then preprocessed in Python 3 using Scanpy [7] (v1.9.3). This preprocessing involved importing data with 'sc.read_h5ad', performing total normalization with 'sc.pp.normalize_total', and applying logarithmic transformation with 'sc.pp.log1p'. UMAP plots and violin plots displaying gene expression levels across different groups were generated using Scanpy's 'sc.pl.umap' and 'sc.pl.violin' functions. Gene expression analyses across groups were visualized using dot plots and heatmaps. These were processed with the AverageExpression function in Seurat, producing a standardized data frame with scaled gene expression levels. For further visualization and to export heatmaps or bubble plots illustrating dynamic gene expression patterns, tools such as pandas, numpy, matplotlib, and plotnine were used. Additionally, to represent quantitative data and the distribution of DEGs across groups, we created plots with the plotnine package in Python 3, utilizing the MAplot function.

**Web server implementation**

The SPARK webserver was developed using Python 3 and Java 17. Data storage is managed via PostgreSQL on the server host. The interactive interface was built with Responsive Vue3 and HTML. The platform is hosted on a Nginx web server and can be accessed online at <http://spark.guolab.info>.

**Reference**

1. Chen, Tingting, Xu Chen, Sisi Zhang, Junwei Zhu, Bixia Tang, Anke Wang, Lili Dong, et al. 2021. “The Genome Sequence Archive Family: Toward Explosive Data Growth and Diverse Data Types.” *Genomics Proteomics Bioinformatics* 19: 578-583. <https://doi.org/10.1016/j.gpb.2021.08.001>

2. Members, Cncb-Ngdc, Partners. 2024. “Database Resources of the National Genomics Data Center, China National Center for Bioinformation in 2024.” *Nucleic Acids Research* 52: D18-D32. <https://doi.org/10.1093/nar/gkad1078>

3. Stuart, Tim, Andrew Butler, Paul Hoffman, Christoph Hafemeister, Efthymia Papalexi, William M 3rd Mauck, Yuhan Hao, Marlon Stoeckius, Peter Smibert, Rahul Satija. 2019. “Comprehensive Integration of Single-Cell Data.” *Cell* 177: 1888-1902 e1821. <https://doi.org/10.1016/j.cell.2019.05.031>

4. Korsunsky, Ilya, Nghia Millard, Jean Fan, Kamil Slowikowski, Fan Zhang, Kevin Wei, Yuriy Baglaenko, Michael Brenner, Po-Ru Loh, Soumya Raychaudhuri. 2019. “Fast, sensitive and accurate integration of single-cell data with Harmony.” *Nature Methods* 16: 1289-1296. <https://doi.org/10.1038/s41592-019-0619-0>

5. Aran, Dvir, Agnieszka P Looney, Leqian Liu, Esther Wu, Valerie Fong, Austin Hsu, Suzanna Chak, et al. 2019. “Reference-based analysis of lung single-cell sequencing reveals a transitional profibrotic macrophage.” *Nature Immunology* 20: 163-172. <https://doi.org/10.1038/s41590-018-0276-y>

6. Van de Sande, Bram, Christopher Flerin, Kristofer Davie, Maxime De Waegeneer, Gert Hulselmans, Sara Aibar, Ruth Seurinck, et al. 2020. “A scalable SCENIC workflow for single-cell gene regulatory network analysis.” *Nature Protocols* 15: 2247-2276. <https://doi.org/10.1038/s41596-020-0336-2>

7. Wolf, F Alexander, Philipp Angerer, Fabian J Theis. 2018. “SCANPY: large-scale single-cell gene expression data analysis.” *Genome Biology* 19: 15. <https://doi.org/10.1186/s13059-017-1382-0>

**Supplementary figures**

**Figure S1.** **Cell distribution information.** The diagram showing the distribution of testicular cell types. SSC, spermatogonial stem cells; S’gonia, spermatogonia; S’cytes, spermatocytes; S’tids, spermatids; TPC, testicular peritubular cells; SMC, smooth muscle cell; EC, endothelial cell.

**Figure S2.** **Atlas Page.** UMAP plots showing the distribution of different datasets in human testes. SSC, spermatogonial stem cells; S’gonia, spermatogonia; S’cytes, spermatocytes; S’tids, spermatids; TPC, testicular peritubular cells; SMC, smooth muscle cell; EC, endothelial cell; KS, Klinefelter syndrome; AZFa/c, Y chromosome azoospermia factor A/C region microdeletion; MO, Mumps orchitis; iNOA, idiopathic non-obstructive azoospermia; yr, years old.

**Figure S3.** **Comparison analysis in the SPARK. (A).** MA plot showing the DEGs between Klinefelter syndrome (KS) and adult cell types. Red/blue indicate up-regulated/down-regulated significant (padj<0.05) genes; **(B).** Violin plots showing the expression of representative genes in different groups and cell types. SSC, spermatogonial stem cells; S’gonia, spermatogonia; S’cytes, spermatocytes; S’tids, spermatids; TPC, testicular peritubular cells; SMC, smooth muscle cell; EC, endothelial cell; yr, years old.

**Figure S4. The process of analysis pipeline for human testicular scRNA-seq datasets in the SPARK.**
